# Quantification of Intact O-Glycopeptides on Haptoglobin in Sera of Patients With Hepatocellular Carcinoma and Liver Cirrhosis

**DOI:** 10.3389/fchem.2021.705341

**Published:** 2021-07-14

**Authors:** Hong Shu, Lei Zhang, Yiwei Chen, Yijie Guo, Limin Li, Fanghua Chen, Zhao Cao, Guoquan Yan, Chunlai Lu, Chao Liu, Shu Zhang

**Affiliations:** ^1^ Liver Cancer Institute, Zhongshan Hospital, and Key Laboratory of Carcinogenesis and Cancer Invasion (Ministry of Education), Fudan University, Shanghai, China; ^2^ Department of Clinical Laboratory, Cancer Hospital of Guangxi Medical University, Nanning, China; ^3^ Institutes of Biomedical Sciences, Fudan University, Shanghai, China; ^4^ Department of Thoracic Surgery, Zhongshan Hospital, Fudan University, Shanghai, China; ^5^ Beijing Advanced Innovation Center for Big Data-Based Precision Medicine, School of Medicine and Engineering, Beihang University, Beijing, China; ^6^ Department of Clinical Laboratory, First Affiliated Hospital of Guangxi Medical University, Nanning, China

**Keywords:** haptoglobin, O-glycosylation, mass spectrometry, lectin, hepatocellular carcinoma

## Abstract

Haptoglobin (Hp) is one of the acute-phase response proteins secreted by the liver, and its aberrant N-glycosylation was previously reported in hepatocellular carcinoma (HCC). Limited studies on Hp O-glycosylation have been previously reported. In this study, we aimed to discover and confirm its O-glycosylation in HCC based on lectin binding and mass spectrometry (MS) detection. First, serum Hp was purified from patients with liver cirrhosis (LC) and HCC, respectively. Then, five lectins with Gal or GalNAc monosaccharide specificity were chosen to perform lectin blot, and the results showed that Hp in HCC bound to these lectins in a much stronger manner than that in LC. Furthermore, label-free quantification based on MS was performed. A total of 26 intact O-glycopeptides were identified on Hp, and most of them were elevated in HCC as compared to LC. Among them, the intensity of HYEGS ^316^TVPEK (H1N1S1) on Hp was the highest in HCC patients. Increased HYEGS ^316^TVPEK (H1N1S1) in HCC was quantified and confirmed using the MS method based on ^18^O/^16^O C-terminal labeling and multiple reaction monitoring. This study provided a comprehensive understanding of the glycosylation of Hp in liver diseases.

## Introduction

Hepatocellular carcinoma (HCC) is one of the leading causes of cancer-related deaths, especially in China. The risk of HCC development depends on many factors such as hepatitis B virus (HBV) infection, and it causes liver complications and liver cirrhosis (Yang et al., 2019). Alpha-fetoprotein (AFP) is the most widely used diagnostic serum biomarker for HCC, however, it has low sensitivity. Glycosylation was reported to be related to most serum tumor biomarkers (Zhu et al., 2019a; Yang et al., 2019). In our previous study, the fucosylated N-glycans of haptoglobin (Hp) were found to be increased in HCC patients (Zhang et al., 2011; Zhang et al., 2013).

As an acute-phase protein mainly synthesized in the liver, Hp comprises heavy chains (*β*,∼ 40 kDa) and light chains (*α*_1_, ∼ 9.1 kDa)/(*α*_2_, ∼ 16 kDa). Its function is to bind and transport free hemoglobin to degrade and recycle iron in the liver (Zhang et al., 2016). Hp contains four N-glycosylation sites, and most of the studies focused on its N-glycosylation, for example, N-glycosylation site occupancy and site-specific N-glycoforms in liver diseases (Zhang et al., 2012). However, limited studies on O-glycans of Hp have been reported. The presence of mono- and disialyl core type 1 O-glycans was reported in prostate cancer Hp (Fujimura et al., 2008).

Development in mass spectrometry (MS) and software has enabled the characterization and quantification of intact glycopeptides in complex biological matrixes. Zhu et al. have identified and quantified N-glycopeptides of Hp based on electron-transfer higher-energy collision dissociation (ET-hcD), MS/MS fragmentation, and Byonic software (Zhu et al., 2019b). We have previously used a glycopeptide method based on ^18^O/^16^O C-terminal labeling and multiple reaction monitoring (MRM) to quantify N-glycopeptides of the 40-kDa band in liver diseases (Zhang et al., 2019).

For this study, serum Hp was purified from patients with HCC and liver cirrhosis (LC), respectively. First, five lectins with the specificity to recognize Gal or GalNAc monosaccharides were used to determine whether Hp could bind. Furthermore, label-free quantification for intact glycopeptide was performed to discover O-glycosylation on Hp. The potential O-glycosylation features were confirmed using the MS quantification method based on ^18^O/^16^O C-terminal labeling and MRM.

## Materials and Methods

### Preparation of Specimens and Purification of Serum Haptoglobin

The serum samples were obtained from the Cancer Hospital of Guangxi Medical University and approved by the Institution Ethics Committee of the Cancer Hospital of Guangxi Medical University (LW2019043). All participants had signed an informed consent form. A summary of pathological patients’ data is given in Table 1. Individuals with autoimmune diseases were excluded from this study. All serum samples were collected using a standard protocol and stored at −80°C until use. For lectin blot analysis and MS quantification based on ^18^O/^16^O C-terminal labeling, pooled serum samples were used. For label-free MS detection and MRM, individual serum samples were chosen. The purification of Hp was performed according to previous reports (Okuyama et al., 2006).

**TABLE 1:** Clinical characteristics of liver cirrhosis and HCC patients.

Group	Set 1 (lectin; ^16^O/^18^O labeling)		Set 2 (label free)		Set 3 (MRM)	
	LC	HCC	LC	HCC	LC	HCC
Number	60	60	7	7	12	12
Age	47.40 ± 11.73	49.65 ± 11.55	48.57 ± 8.02	47.4 ± 10.27	50.13 ± 8.55	51.0 ± 10.29
Gender (M/F)	50/10	55/5	6/1	6/1	8/4	11/1
ALT, U/L	30 (5–216)	42 (12–428)	32 (17–121)	36 (15–199)	27 (17–121)	53 (15–199)
AST, U/L	35 (8–182)	38 (15–355)	33 (19–145)	50 (24–220)	34 (19–145)	43 (24–220)
HBsAg^+^(%)^a^	100	100	100	100	100	100
AFP, ng/mL	6.00 (1.2–65.1)	153.3 (1.6–60500.0)	3.86 (1.35–170.0)	94.1 (1.41–60500.0)	3.26 (1.35–170)	73.76 (1.41–60500)

ALT, alanine aminotransferase; AST, aspartate transaminase; HbsAg, hepatitis B surface antigen; AFP, alpha fetoprotein.

a Normal results of HBsAg+ (%) are negative or nonreactive, meaning that no HBsAg was found; if the test is positive or reactive, it may mean active infection with HBV or chronic hepatitis B infection.

### Lectin Blot Analysis

Purified Hp of 60 *μ*l of sera (pooled from 20 patients, 3 *μ*l per patient) from LC and HCC, respectively, was analyzed by lectin blot according to the previous description (Shu et al., 2011). Five biotinylated lectins (Vector Laboratories, Burlingame, CA) including *Amaranthus caudatus* lectin (ACA, 1 *μ*g/ml), *Griffonia simplicifolia* lectin I (GSI-L, 1 *μ*g/ml), Jacalin (JAC, 1 *μ*g/ml), *Vicia villosa* lectin (VVA, 1 *μ*g/ml), and *Wisteria floribunda* lectin (WFA, 2 *μ*g/ml) were incubated for 45 min, respectively. After washing and incubating with horseradish peroxidase Avidin D (Vector Laboratories, Burlingame, CA), the bands were developed using chemiluminescence detection reagents (GE Healthcare, Piscataway, NJ).

### In-Gel Digestion and O-Glycopeptide Enrichment

Purified Hp was obtained from an equal volume of each sample and subjected to SDS-PAGE. The bands of the α_1_, α_2_, and *β* chains were excised, destained, reduced, and alkylated. PNGase F treatment (enzyme:substrate = 1:20, v/w) was performed to release N-glycans from the gel at 37°C overnight. Then, the gel particles were incubated in sequencing grade trypsin (Promega, Madison, WI) at 37°C overnight for in-gel digestion (enzyme:substrate = 1:50, w/w). Subsequently, the peptides were extracted three times with 50% ACN and 0.1% trifluoroacetic acid and lyophilized. The O-glycopeptides were enriched in accordance with the manufacturer’s protocol for the glycopeptide enrichment kit (Novagen, Darmstadt, Germany).

### Nano-Liquid Chromatography Tandem Mass Spectrometry

The experiments were performed using an Orbitrap Fusion MS (Thermo Fisher Scientific, Bremen, Germany). Purified Hp from 30 μl sera of LC and HCC, respectively, was used as starting material. Freeze-dried peptides were redissolved with 10 μl solvent A [solvent A: 0.1% formic acid (FA) and water solution], and were directly injected into the analytical column (Acclaim PepMap C18, 75 μm × 25 cm). To achieve sufficient separation, the samples were eluted for 90 min with the following linear gradient: 1% solvent B (solvent B: 0.1% FA in ACN) to 25% mobile phase B in 60 min, from 25 to 45% mobile phase B in 20 min, followed by an increase to 90% mobile phase B in 1 min, which is maintained for 3 min and finally re-equilibrated for 6 min at 1% B. The flow rate of the column was 300 nL/min, and the column temperature was maintained at RT. Ions with charge states between 2 + and 6+ were sequentially fragmented by HCD with a stepped collision energy of 20, 30, and 40%.

### O-Glycopeptide Identification Using Byonic

The raw files were searched using Byonic (Protein Metrics, San Carlos, CA) against the fasta-containing sp|P00738|HPT_HUMAN (the UniProt human haptoglobin database) due to the searching capacity. A human O-glycan database that included 70 human O-glycans was used for searching. Specific parameters were as follows: the tolerances were 20 ppm for precursors and 0.05 Da for fragment ions, respectively. Up to two missed cleavages were allowed. The Carbamidomethyl/+57.022 Da of C was set as a fixed modification, while Oxidation/+15.995 Da of M and Deamidated/+0.98 Da of N were set as the variable modifications. Quality control was performed by FDR estimation, and those below the criterion of a 1% FDR were considered. The mass range for the search was set between 350 and 5,000 Da. The predicted O-glycosylation sites were also considered. The NetOGlyc 4.0 server was used to predict O-glycosylation sites on Hp in humans (http://www.cbs.dtu.dk/services/NetOGlyc). Identification of MS/MS spectra was presented using the gLabel software tool (Liu et al., 2017), which contributes toward manual verification.

### Label-Free Quantification for O-Glycopeptide

For the MS^1^ level-based label-free method, the normalization was performed using pQuant (Liu et al., 2014). It first obtains the original intensity matrix X (Eq. 1) and then calculates the normalized intensity according to each sample. For each intensity X_i,j_, pQuant divides it by the mean intensity of the corresponding sample and calculates the logarithm to get X_mean_process (Eq. 2). Then, the median intensity of each sample processed by the mean value is subtracted from the intensity just calculated, and the intensity of the whole sample is shifted so that the median is 0 (Eq. 3). Finally, the normalized intensity is obtained as follows:

$$X=\left(x_{1},x_{2},\ldots ,x_{n}\right),n=sample\;number\;,$$ (1)

$$x\_mean\_process_{i,j}=\log\left(\;x_{i,j}/\;\frac{1}{m}\times \sum_{l=1}^{m}x_{i,l}\right)\;,\text{m}=\text{peptide number,}$$ (2)

$$Normalized\;Intensity\;x_{i,j}=x\_mean\_process_{i,j}-median(x\_mean\_process{,}_{i}).$$ (3)

After normalization, the intensities of intact glycopeptide were calculated using the formula; for example, intensity of O-glycopeptide 1 in LC1 = (normalized intensity of O-glycopeptide 1 in LC1) × adjust ratio = (normalized intensity of O-glycopeptide 1 in LC1) × [(normalized intensity of O-glycopeptide 1 in LC1)/(all normalized intensities of O-glycopeptides in LC1)].

### O-Glycopeptide Quantification Using ^18^O/^16^O Labeling

Purified Hp of 300 μl sera (pooled from 20 patients, 15 μl per patient) from LC and HCC was obtained, respectively. After in-gel digestion and O-glycopeptide enrichment, immobilized trypsin (Thermo Scientific, Rockford, IL) was added (enzyme:substrate = 1:5 v:w) and freeze-dried. The two freeze-dried powders were resolubilized with 20% ACN in H_2_^16^O/H_2_^18^O (97%, Cambridge Isotope Laboratories, Andover, MA), respectively, at 37°C for 24 h. The reaction was quenched by adding 1 μl FA, and the supernatant was collected using centrifuge columns (Pierce, Rockford, IL). The ^16^O- and ^18^O-fractions were redissolved with 5 μl solvent A, respectively (solvent A: 0.1% FA in water), and combined, isolated by nano-LC, and detected by online electrospray tandem MS. ^18^O/^16^O labeled O-glycopeptides were identified using Byonic and quantified using pQuant. The ^18^O/^16^O glycopeptide ratio is based on a pair of isotope chromatograms with the least interference to exclude the overlap of the ^16^O and ^18^O isotopic peaks. pQuant usually chooses the monoisotopic peak from light labeling because it is usually the least interfered peak for light labeling. Then, due to different interference conditions, a different peak from heavy labeling was considered. If the monoisotopic peak of heavy labeling was chosen, the algorithm in our previous study (Zhang et al., 2019) was used. The denominator of the formula, such as $I_{2}+I_{4}-\left(\frac{M_{2}}{M_{0}}\right)I_{2}-\left[\left(\frac{M_{2}}{M_{0}}\right)+\left(\frac{M_{4}}{M_{0}}\right)-{\left(\frac{M_{2}}{M_{0}}\right)}^{2}\right]I_{0}$, subtracts the light labeling effect from heavy labeling. If the other peak from heavy labeling was chosen (not the monoisotopic or the first peak), all the isotopic chromatograms were normalized using pQuant. The normalized chromatograms of the light peptide are *L*_1_ = *p* ^*l*^_1_ (Σ*t* ^*l*^_i_/*t* ^*l*^_l_), *L*_2_ = *p* ^*l*^_2_ (Σ*t* ^*l*^_i_/*t* ^*l*^_2_), and *L*_N_ = *pl*_N_ (Σ*t* ^*l*^_i_/*t* ^*l*^_N_). The normalized chromatograms of the heavy peptide are *H*_1_ = *p* ^*h*^_1_ (Σ*t* ^*h*^_i_/*t* ^*h*^_1_), *H*_2_ = *p* ^*h*^_2_ (Σ*t* ^*h*^_i_/*t* ^*h*^_2_), and *H*_M_ = *p* ^*h*^_M_(Σ*t* ^*h*^_i_/*t* ^*h*^_M_). Once normalized, the monoisotopic peak from the light peptide and the selected peak from the heavy labeling can be used to calculate the H/L ratio of the glycopeptide.

### Multiple Reaction Monitoring Validation

Purified Hp was obtained from 100 μl of serum of each sample, and a total of 24 patients were enrolled (12 HCC patients and 12 LC patients). After digestion overnight with trypsin, the samples were diluted in 2% ACN and 0.1% FA and ionized using an Ultimate 3,000 HPLC (150 × 2.1 mm, 3 μm, 100 Å column, Thermo, Framingham, United States) coupled with a 6,500 QTRAP MS (ABSciex, Waltham, United States). Peptide separation of the individual samples was achieved using the following 30-min gradient for flow rates of 200 μL/min, with solvent A (0.1% FA in water) and solvent B (0.1% FA in ACN): 5% B for 1 min, 5%–50% B for 13 min, 80% B for 0.5 min, and 50% B for 7.5 min. The optimum transitions and one unique peptide of Hp were selected for each glycopeptide/peptide for MRM monitoring. Analyzer parameters were optimized for each peptide/transition pair to ensure maximum selectivity. Both Q1 and Q3 resolutions were chosen as “Unit” (±0.7 Da). The acquired MRM wiff files were analyzed using Skyline software. The precursor ion of the O-glycopeptide was chosen, and the product ions were used to determine the peak area for the O-glycopeptide.

### Statistical Analysis

All data and graphs were generated using GraphPad Prism 7.0 software (GraphPad Software, Inc.). Statistical comparisons were carried out using the *t*-test, and *p* values < 0.05 presented as statistically significant. Lectin blot and MRM data were evaluated using nonparametric Mann–Whitney *U* tests.

### Data Availability Statement

The mass spectrometry data have been deposited to the ProteomeXchange Consortium (http://proteomecentral.proteomexchange.org) *via* the iProX partner repository (Ma et al., 2019) with the dataset identifier PXD023447.

## Results and Discussion

### O-Glycosylation Status of Haptoglobin by Lectin Blot Analysis

Hp is a glycoprotein produced in the liver that is secreted into the blood. Lectins are defined as carbohydrate-binding proteins and have biotechnological implications. Lectin-based methods were developed for the detection of fucosylated Hp (Miyoshi and Kamada. 2016; Shang et al., 2017), and they may serve as a cancer biomarker for clinical application. Fucosylation is one of the most important types of glycosylation in cancer and inflammation, for example, the core-fucosylated T-cell receptor was necessary for T-cell signaling and production of inflammatory cytokines (Fujii et al., 2016). However, limited studies on O-glycans of Hp have been reported. O-glycosylation of Hp was found in pigs with PCV2-SD infection, and the presence of mono- and disialyl core type 1 O-glycans of Hp was found in prostate cancer (Fujimura et al., 2008; Marco-Ramell et al., 2014). Here, lectin blotting was used to determine the O-glycosylation of Hp in liver diseases.

**FIGURE 1:**
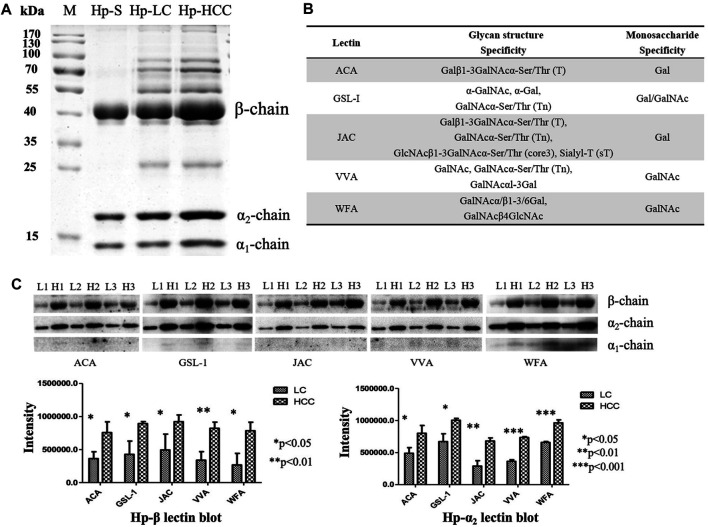
Purification of Hp and its O-glycosylation status by lectin blot analysis. **(A)** Purified Hp from the sera of LC and HCC patients. M, marker; Hp-S, Hp standard; Hp-LC, purification of Hp from LC patients; Hp-HCC, purification of Hp from HCC patients. **(B)** Carbohydrate-binding specificities of five lectins (ACA, GSL-1, JAC, VVA, and WFA). **(C)** Lectin blot was applied to reveal the O-glycan levels of Hp. Hp from an equal volume of LC and HCC sera was used, and three biological repeats were performed. *p*-value of less than 0.05 showing statistical significance using nonparametric Mann–Whitney *U* tests. L, LC; H, HCC.

First, Hp was purified from an equal volume of LC and HCC sera, respectively (Figure 1A), and the gel bands were cut, digested, and determined by LC-MS/MS. The corresponding MS results confirmed that these proteins in the gels were Hp (Supplementary Figure S1). Then, five lectins including ACA, GSL-1, JAC, VVA, and WFA were chosen to reveal the potential O-glycosylation status of Hp in LC and HCC. The monosaccharide-binding specificities of these lectins are presented in Figure 1B. Lectin blot analysis showed that the *β*- and *α*_2_-chains of Hp in HCC had a higher binding ability with regard to these lectins than those in cirrhosis (Figure 1C and Supplementary Material). Both predicted and experimentally determined O-glycosylation sites were nearly in accordance with those already known O-glycosylation sites, such as fibrinogen, *α*-2-HS-glycoprotein, and so on (Hoffmann et al., 2016). In this study, according to the Hp sequence, O-glycosylation at four Thr residues (Thr67, 126, 317, and 323) and one Ser residue (Ser316) has been predicted using the NetOGlyc 4.0 server (Steentoft et al., 2013). The results indicated the possible presence of O-glycans on Hp, and it also displayed differences in liver diseases.

### Intact O-Glycopeptides of Haptoglobin in Liver Cirrhosis and Hepatocellular Carcinoma

**FIGURE 2:**
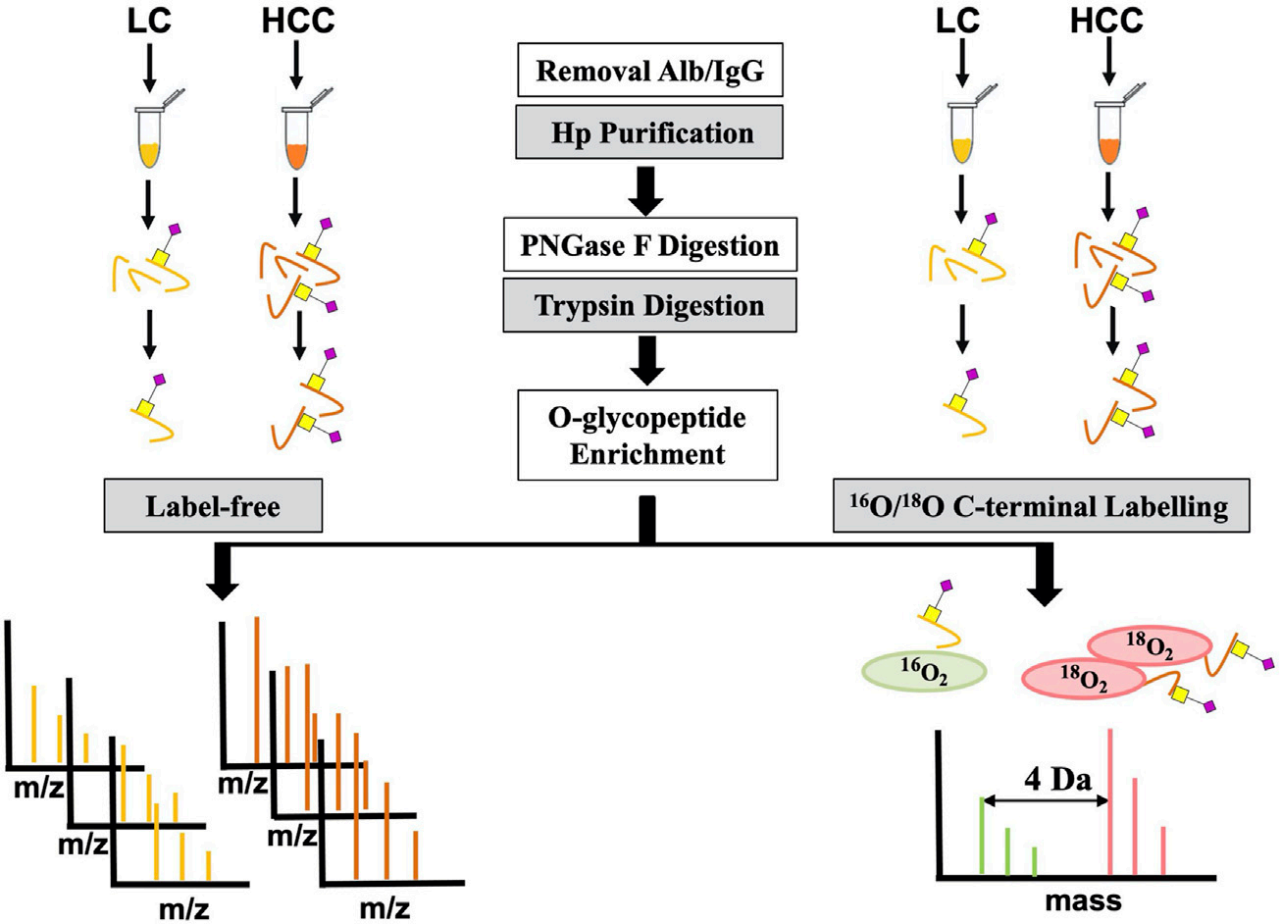
Workflow for label-free and labeling quantification of O-glycopeptides on Hp.

To further confirm its O-glycosylation in HCC, both the label-free and labeling methods based on MS were applied (Figure 2). A total of seven HCC and seven LC serum patients were first used for label-free quantification. For this method, serum Hp was purified from an equal volume of patients with LC and HCC. Then, purified Hp was treated with PNGase F and trypsin. After glycopeptide enrichment, they were subjected to LC-MS/MS analysis, respectively. All spectra raw files were automatically identified using Byonic.

**FIGURE 3:**
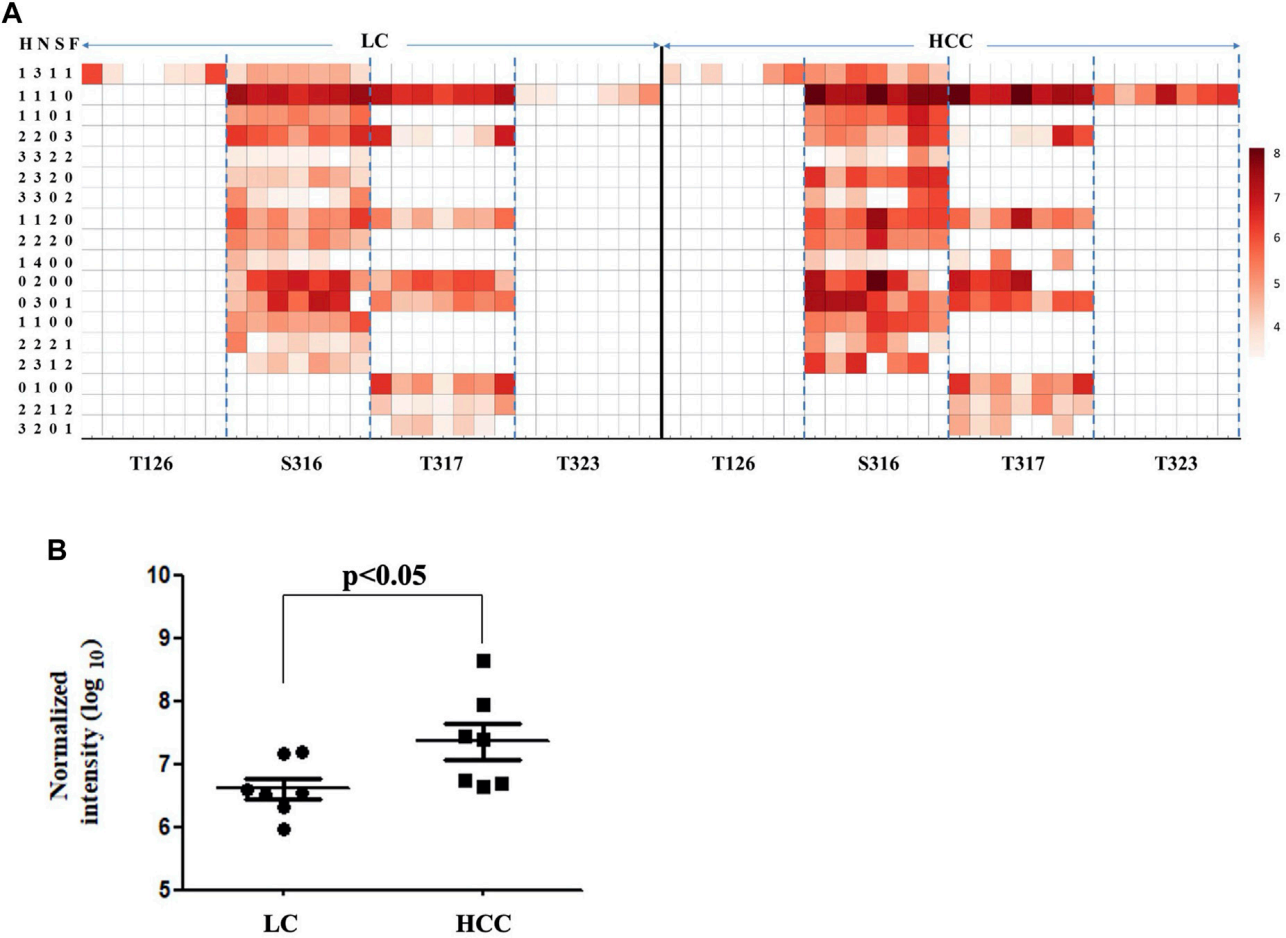
Abundance of Hp O-glycopeptides in LC and HCC. **(A)** Intensity distributions of Hp O-glycopeptides in seven HCC and seven LC patients. The *X*-axis represents identified O-glycosylation sites of Hp. The *Y*-axis represents the intensity (Log10 conversion) of different glycoforms in Hp. **(B)** Label-free quantification of HYEGS ^316^TVPEK (H1N1S1) of Hp and the difference was statistically significant.

As shown in Figure 3A, 26 intact O-glycopeptides on four O-glycosylation sites (Thr126, 317, 323, and Ser316) were identified on the Hp protein and corresponded to 18 types of glycan compositions. Most of them were elevated in HCC as compared to LC. As shown in Supplementary Table S1, 57.69% of the changed glycopeptides were observed in seven patients and 96.15% in at least four patients. In addition, the majority of the glycoforms were located on Ser316 and Thr317, while one glycoform was located on Thr126 and Thr323. Moreover, one glycoform (H1N4) on Thr317 could be detected in HCC; however, this was absent in cirrhosis. Among these O-glycopeptides on Hp, the intensity of HYEGS ^316^TVPEK (H1N1S1) was the highest, and it was significantly increased in HCC patients (Figure 3B, *p* < 0.05). The glycan composition of this O-glycopeptide was core 1 type O-glycans with one NeuAc residue.

Aberrant fucosylation and sialylation have been reported in various cancers (Verhelst et al., 2020). As one of the acute-phase response proteins, Hp contains four N-glycosylation sites (Asn184, 207, 211, and 241), and its fucosylated N-glycans were reported in multiple cancer types (Takahashi et al., 2016). In this study, the sialylation alteration of O-glycans on Hp provided clues to the comprehensive understanding of its glycosylation in cancers.

### Intact O-Glycopeptide HYEGS ^316^TVPEK (H1N1S1) on Haptoglobin

**FIGURE 4:**
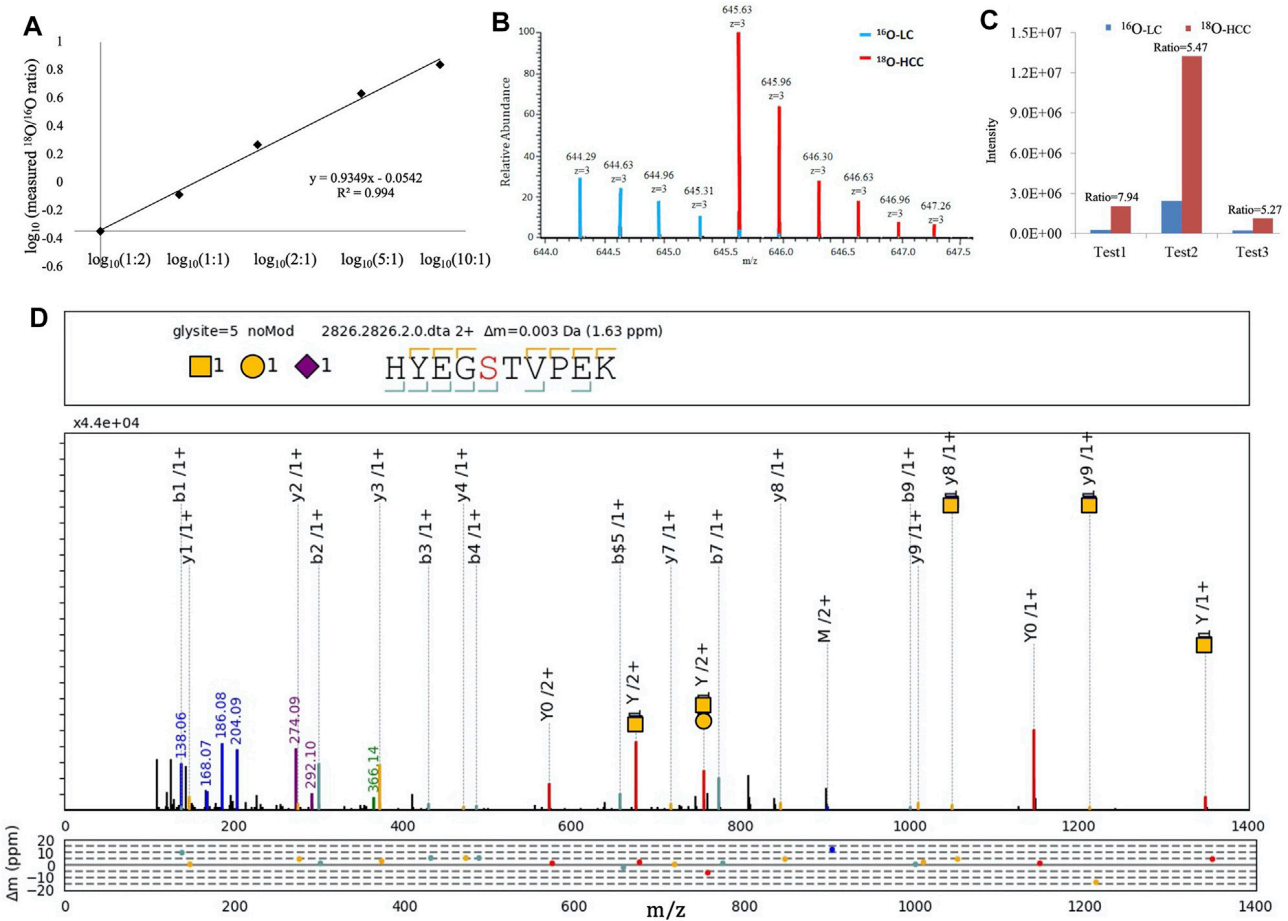
^16^O/^18^O C-terminal labeling for quantification of Hp O-glycopeptides. **(A)** Series of theoretical ratios of Hp O-glycopeptide standard (1:2, 1:1, 2:1, 5:1, and 10:1) was applied, and experimental ratios showed good linearity with a correlation coefficient (*R* ^2^) approximate of 0.99. **(B)** Representative MS^1^ spectrum of HYEGS ^316^TVPEKK (H1N1S1). **(C)** Three biological repeats were performed, and the average fold change (HCC/LC) of this O-glycopeptide was about 6.2. **(D)** Representative MS^2^ spectrum was shown using the gLabel software tool. The O-glycosylation site is “S.” The glycan symbols are as follows: purple diamond for sialic acid (S), yellow circle for hexose (H), and yellow square for N-acetylhexosamine (N). The glycan composition and peptide sequence are in the upper box of each spectrum. The peak annotations are displayed in the middle box containing fragment ions of the glycan part or diagnostic glycan ions (green, blue, and purple), y-ions from glycan fragmentation (red), and the b/y ions from peptide backbone fragmentation (yellow/cyan). Mass deviations in the annotated peaks are displayed in the box below.

We focused on HYEGS ^316^TVPEK (H1N1S1), which had the highest intensity and was significantly increased in HCC patients. The labeling method was applied to confirm this change. For the labeling method, O-glycopeptides from LC were labeled by H_2_^16^O and those from HCC were labeled by H_2_^18^O. There were 4-Da mass shifts between ^16^O- and ^18^O-labeled samples. Labeled samples were pooled and detected by LC-MS/MS. The reproducibility and quantitative accuracy of this labeling strategy were first evaluated using standard Hp. A series of theoretical ratios of standard (1:2, 1:1, 2:1, 5:1, and 10:1) was applied. The O-glycopeptide HYEGS^316^TVPEK (H1N1S1) was identified, and experimental ratios of this O-glycopeptide showed good linearity with a correlation coefficient (*R* ^2^) approximate of 0.99 (Figure 4A). For LC and HCC samples, this O-glycopeptide of Hp was elevated significantly in HCC patients (Figure 4B). Based on three biological replicates, the average fold change (HCC/LC) of this O-glycopeptide was about 6.2 (Figure 4C). Figure 4D shows the representative MS^2^ spectrum of this O-glycopeptide, annotated using the gLabel software tool.

Intact glycopeptide analysis including glycosylation sites and site-specific glycans is crucial for the understanding of glycosylation (Medzihradszky et al., 2015). The degree of O-glycan sialylation was observed to be associated with the pathogenesis of many diseases (Campbell et al., 2001). However, the minor level of O-glycosylation posed a challenge for further analyses of it (Yang et al., 2018; Zhang et al., 2018). Using label-free and labeling quantification, elevated HYEGS ^316^TVPEK (H1N1S1) of Hp in HCC was found in this study.

### Multiple Reaction Monitoring Analyses of HYEGS ^316^TVEPK (H1N1S1)

**FIGURE 5:**
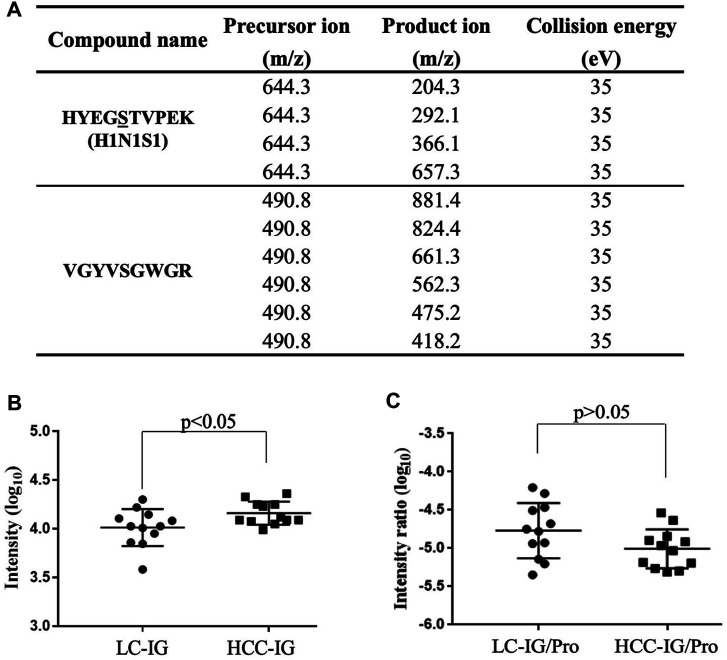
MRM for validation. **(A)** MRM transitions for monitoring the O-glycopeptide and the unique peptide of Hp. **(B)** HYEGS ^316^TVPEK (H1N1S1) of Hp from 12 LC patients and 12 HCC patients was detected, and the difference was statistically significant. **(C)** O-glycopeptide abundance was divided by the unique peptide abundance to separate out the contribution of protein concentration. The result showed that the increased HYEGS ^316^TVEPK (H1N1S1) was caused by elevated protein expression in HCC. LC-IG, the intact O-glycopeptide in LC; HCC-IG, the intact O-glycopeptide in HCC; LC-IG/Pro, the intact O-glycopeptide abundance divided by the protein abundance in LC; HCC-IG/Pro, the intact O-glycopeptide abundance divided by the protein abundance in HCC.

To further confirm elevated HYEGS ^316^TVPEK (H1N1S1) of Hp in HCC, MRM analysis was also applied (Sanda et al., 2013). The target O-glycopeptide HYEGS ^316^TVPEK (H1N1S1) and the unique peptide of Hp were chosen for MRM transitions (Figure 5A). In this study, peak areas of the O-glycopeptide were employed for absolute quantification, and a unique peptide was used to confirm the purification of Hp. Figure 5B shows that this O-glycopeptide was also significantly elevated in HCC patients as compared to LC (*p* < 0.05). Thus, MRM analyses validated this intact O-glycopeptide on Hp which exhibited differences in HCC. We also used the unique peptide of Hp (VGYVSGWGR) as an internal standard, and the O-glycopeptide abundance was divided by the unique peptide abundance to separate out the contribution of protein concentration. The result showed that the increased HYEGS ^316^TVEPK (H1N1S1) was caused by elevated protein expression in HCC (Figure 5C).

Mass spectrometry–based strategies for glycopeptide quantification include isotopic labeling and the label-free method (Delafield and Li, 2021). For the labeling strategy, the quantitative result can be obtained simultaneously by comparing the abundance of the isotopologues. The label-free strategy requires the stability of the production, and it has benefited from the implementation of MRM. For MRM analysis, effective ionization of glycopeptides and the reproducible fragments are important. In our study, MS^1^ level–based label-free quantification was performed first to screen O-glycopeptides. The ^16^O/^18^O-labeled method and MS^2^ level–based MRM were used to quantify the significantly changed O-glycopeptide and to accurately confirm this alteration. All the above results indicated the existence of sialylated O-glycans on Hp. Compared to the LC patients, significantly elevated HYEGS ^316^TVPEK (H1N1S1) of Hp was identified in HCC. More studies are still needed to increase the sensitivity for O-glycosylation analyses and uncover its biological function in liver diseases.

## Conclusion

In the present study, we demonstrated that the intact O-glycopeptide HYEGS ^316^TVPEK (H1N1S1) increased significantly in HCC patients. Changes in protein glycosylation might be used as diagnostic and prognostic markers, as well as targets of therapy for cancer. The aberrant O-glycopeptide on Hp was explored in connection with liver disease, and this study provided clues to a comprehensive understanding of its glycosylation in cancers.

## Data Availability

The datasets presented in this study can be found in online repositories. The names of the repository/repositories and accession number(s) can be found below: the ProteomeXchange Consortium (http://proteomecentral.proteomexchange.org) *via* the iProX partner repository with the dataset identifier PXD023447.
