# Supplementary figures and images for: Quantification of Intact O-Glycopeptides on Haptoglobin in Sera of Patients With Hepatocellular Carcinoma and Liver Cirrhosis
Source: Front Chem. 2021 Jul 14;9:705341. doi: 10.3389/fchem.2021.705341 (PMC8316590; doi:10.3389/fchem.2021.705341)

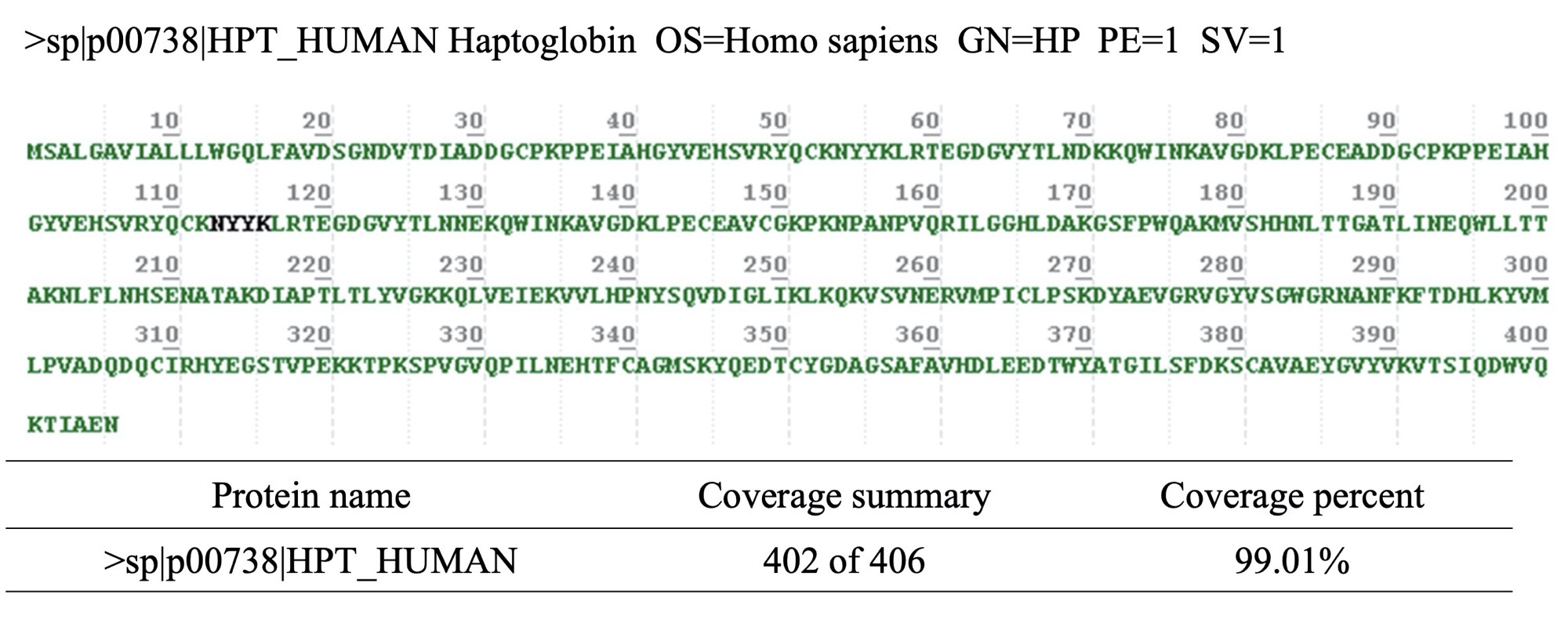

Supplement: Supplementary file 1 [file Image1.JPEG]

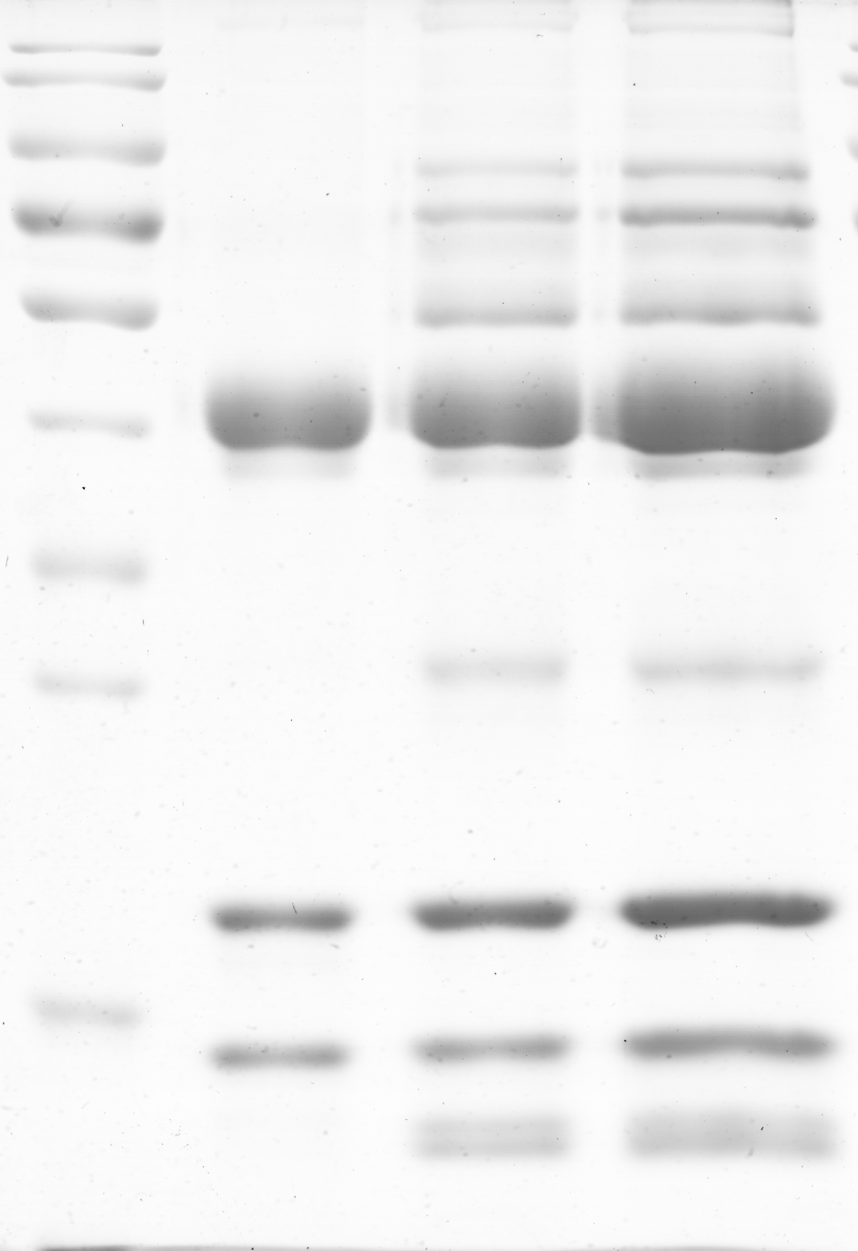

Supplement: Supplementary file 2 [file DataSheet1.ZIP › Figure1A-C/SDS-PAGE.tif]

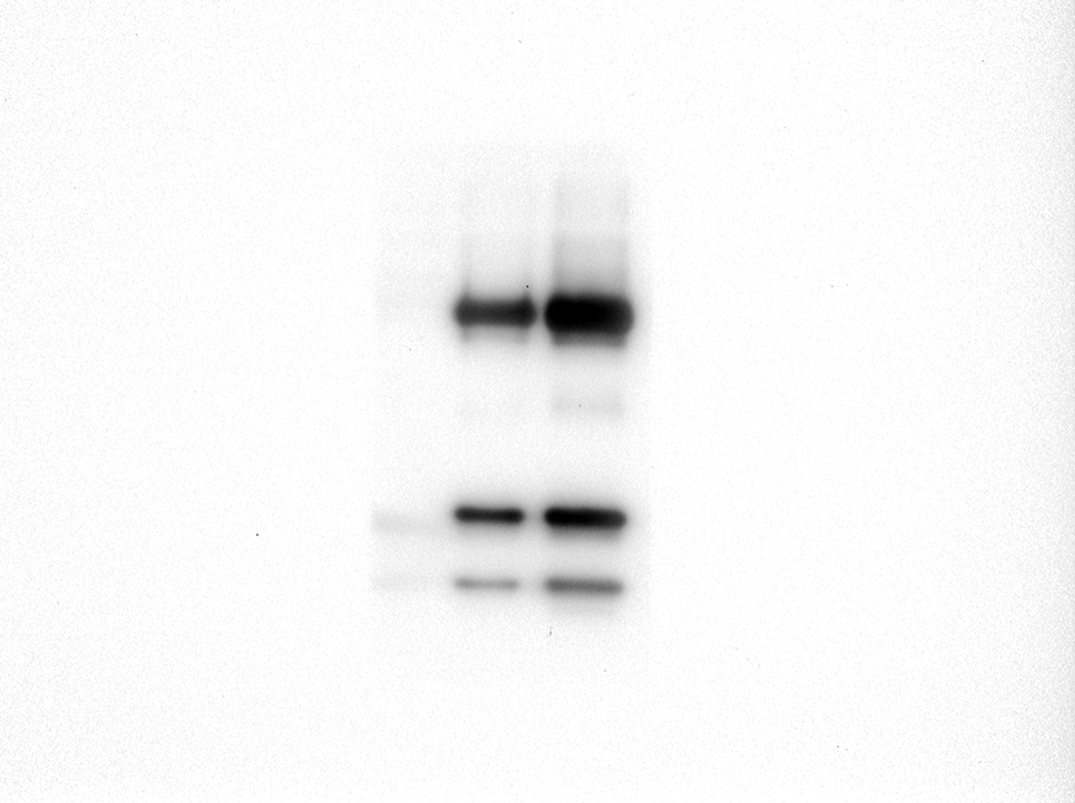

Supplement: Supplementary file 2 [file DataSheet1.ZIP › Figure1A-C/western blot/L2H2-WB.tif]

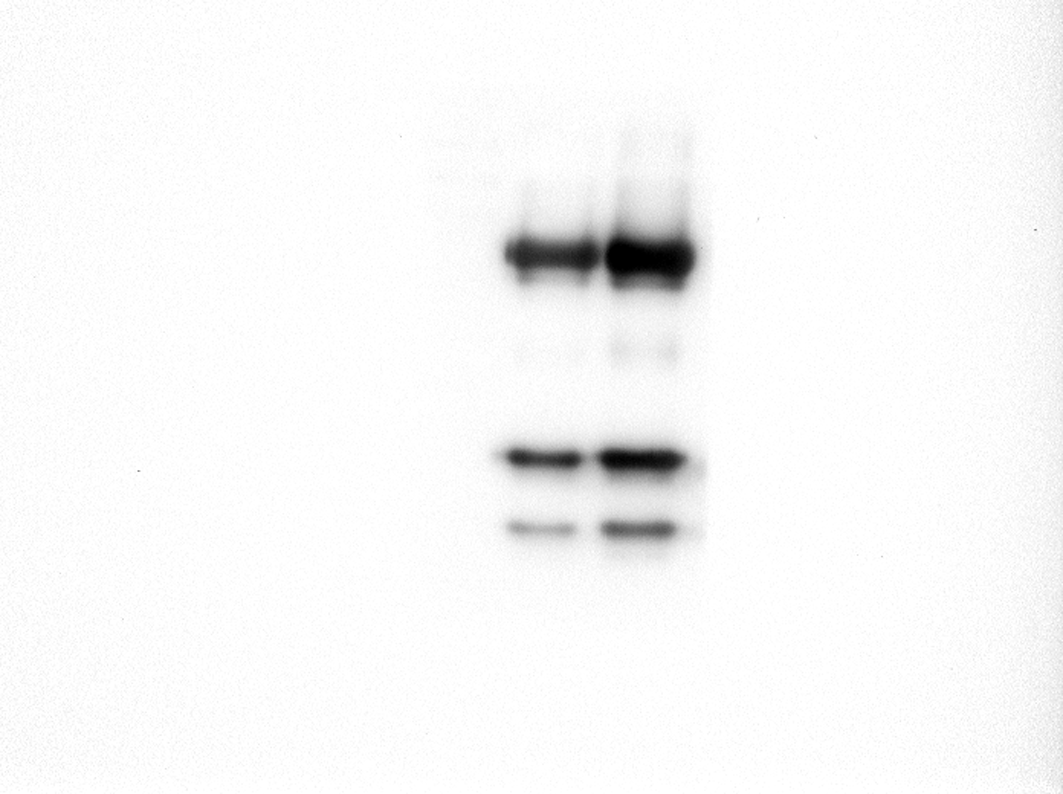

Supplement: Supplementary file 2 [file DataSheet1.ZIP › Figure1A-C/western blot/L1H1-WB.tif]

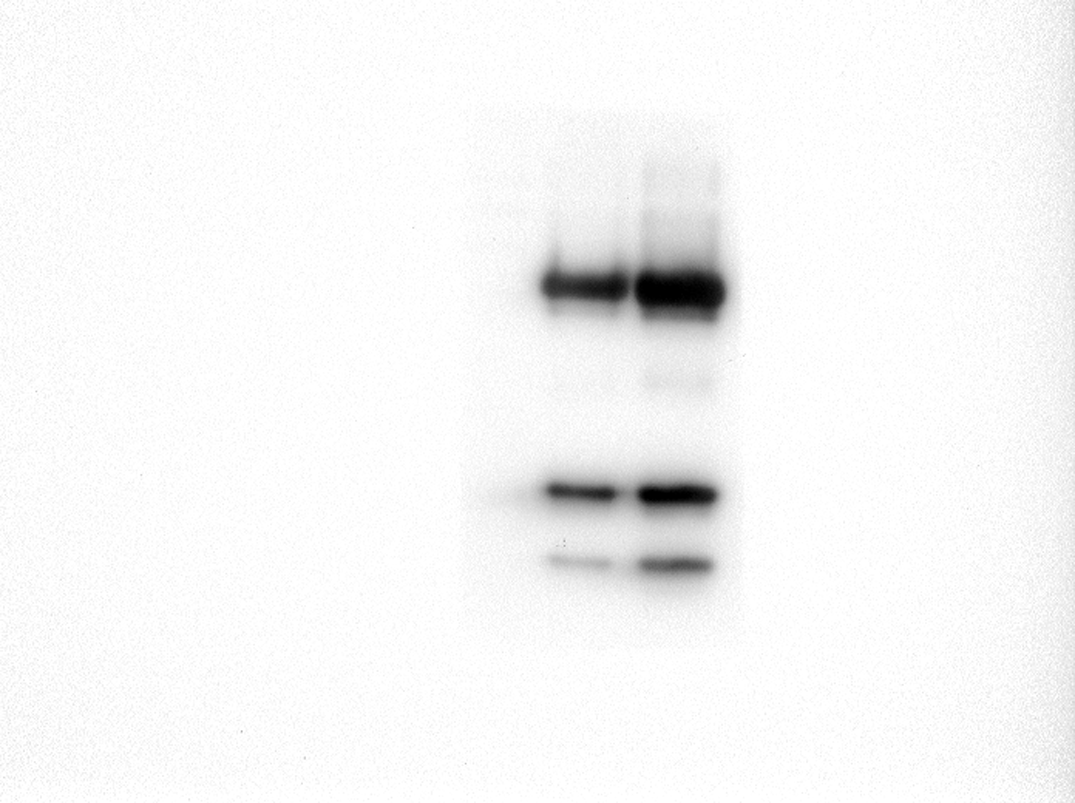

Supplement: Supplementary file 2 [file DataSheet1.ZIP › Figure1A-C/western blot/L3H3-WB.tif]

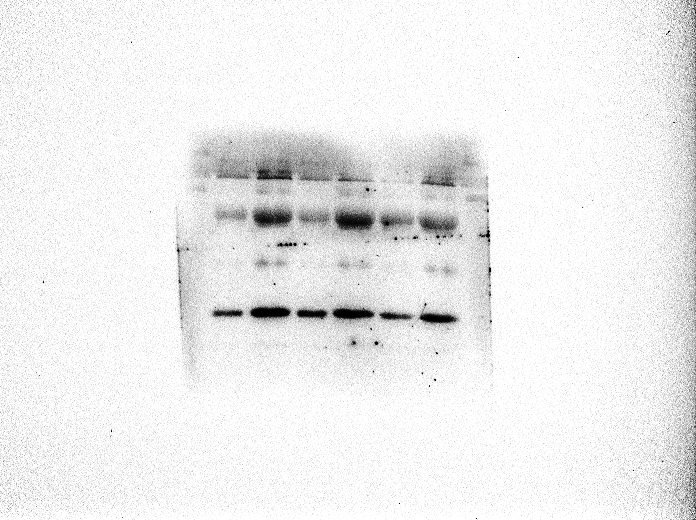

Supplement: Supplementary file 2 [file DataSheet1.ZIP › Figure1A-C/lectin blot/ACA.tif]

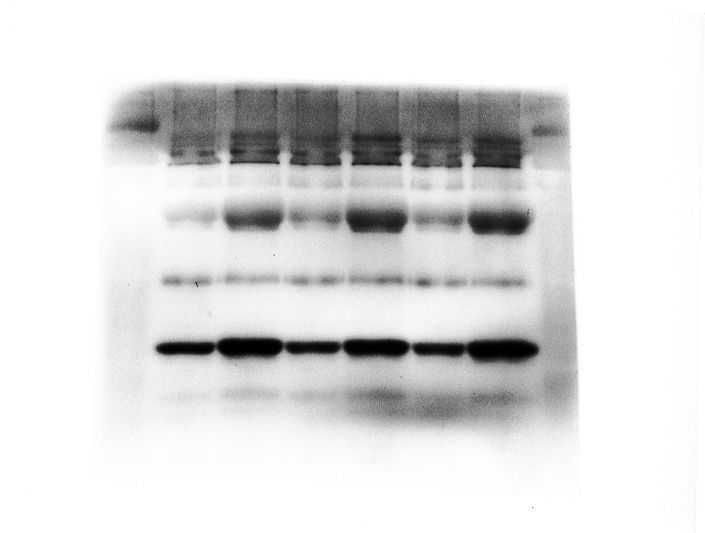

Supplement: Supplementary file 2 [file DataSheet1.ZIP › Figure1A-C/lectin blot/WFA.tif]

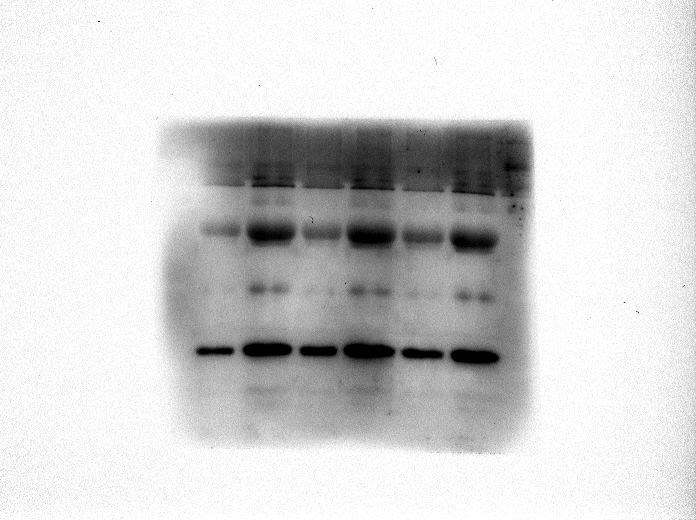

Supplement: Supplementary file 2 [file DataSheet1.ZIP › Figure1A-C/lectin blot/GSL.tif]

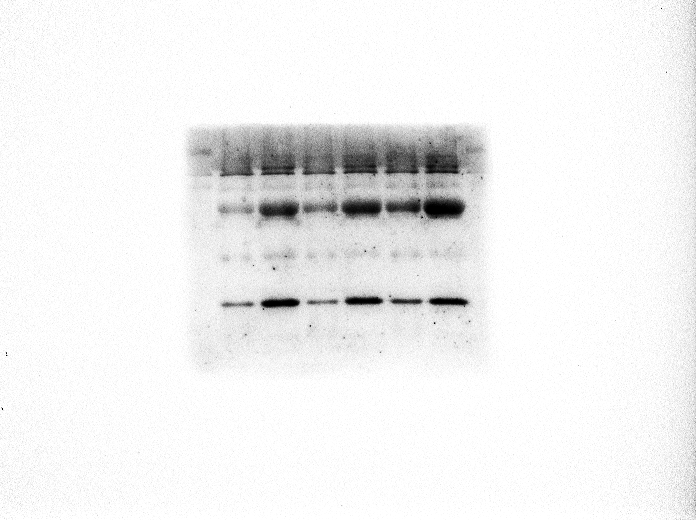

Supplement: Supplementary file 2 [file DataSheet1.ZIP › Figure1A-C/lectin blot/JAC.tif]

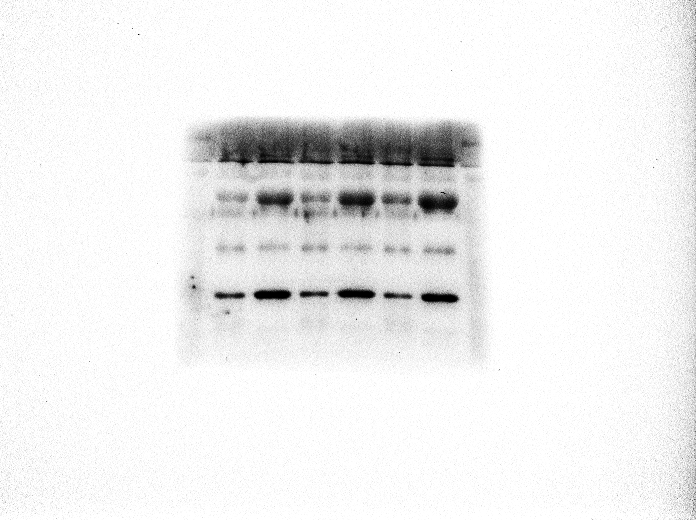

Supplement: Supplementary file 2 [file DataSheet1.ZIP › Figure1A-C/lectin blot/VVA.tif]
